# A quantitative method to detect non-antithrombin-binding 3-*O*-sulfated units in heparan sulfate

**DOI:** 10.1074/jbc.RA120.015864

**Published:** 2020-12-03

**Authors:** Hideo Mochizuki, Hideyuki Futatsumori, Eriko Suzuki, Koji Kimata

**Affiliations:** 1 Central Research Laboratories, Seikagaku Corporation, Higashiyamato, Tokyo, Japan; 2 Multidisciplinary Pain Center, Aichi Medical University, Nagakute, Aichi, Japan

**Keywords:** glycosaminoglycan, heparan sulfate, sulfotransferase, neuron, differentiation, 3-*O*-sulfate, P19 cells, 3OST, heparan sulfate 3-*O*-sulfotransferase, AT, antithrombin, FBS, fetal bovine serum, GlcA, D-glucuronic acid, GlcNAc, *N*-acetyl-D-glucosamine, HexA, hexuronic acid, Hpa-1, human heparanase-1, IdoA, L-iduronic acid, PAPS, 3'-phosphoadenosine 5'-phosphosulfate

## Abstract

Heparan sulfate is synthesized by most animal cells and interacts with numerous proteins *via* specific sulfation motifs to regulate various physiological processes. Various 3-*O*-sulfated motifs are considered to be key in controlling the binding specificities to the functional proteins. One such motif synthesized by 3-*O*-sulfotransferase-1 (3OST-1) serves as a binding site for antithrombin (AT) and has been thoroughly studied because of its pharmacological importance. However, the physiological roles of 3-*O*-sulfates produced by other 3OST isoforms, which do not bind AT, remain obscure, in part due to the lack of a standard method to analyze this rare modification. This study aims to establish a method for quantifying 3-*O*-sulfated components of heparan sulfate, focusing on non-AT-binding units. We previously examined the reaction products of human 3OST isoforms and identified five 3-*O*-sulfated components, including three non-AT-binding disaccharides and two AT-binding tetrasaccharides, as digestion products of heparin lyases. In this study, we prepared these five components as a standard saccharide for HPLC analysis. Together with eight non-3-*O*-sulfated disaccharides, a standard mixture of 13 units was prepared. Using reverse-phase ion-pair HPLC with a postcolumn fluorescent labeling system, the separation conditions were optimized to quantify the 13 units. Finally, we analyzed the compositional changes of 3-*O*-sulfated units in heparan sulfate from P19 cells before and after neuronal differentiation. We successfully detected the 3-*O*-sulfated units specifically expressed in the differentiated neurons. This is the first report that shows the quantification of three non-AT-binding 3-*O*-sulfated units and establishes a new approach to explore the physiological functions of 3-*O*-sulfate.

Heparan sulfate is synthesized by most animal cells as a component of proteoglycans and interacts with numerous proteins such as growth factors, morphogens, receptors, and extracellular matrix proteins to regulate various physiological processes (1). The heparan sulfate molecule is composed of densely sulfated regions connected by mostly nonsulfated regions (2). Most of the interactions between heparan sulfate and functional proteins are thought to occur at the sulfated regions having specific arrangements of sulfation motifs (3). The biosynthesis of heparan sulfate is initiated by the polymerization of alternating D-glucuronic acid (GlcA) and *N*-acetyl-D-glucosamine (GlcNAc) residues to form the repeating disaccharide structure -GlcA-β1,4-GlcNAc-α1,4-. This polymer is then partially *N*-deacetylated/*N*-sulfated and subsequently undergoes 5-epimerization of GlcA to L-iduronic acid (IdoA), 2-*O*-sulfation of hexuronic acid (HexA) residues and 6-*O*-sulfation of glucosamine residues. Then, a rare but functionally important modification, 3-*O*-sulfation of the glucosamine residues, also occurs. These modification processes are generally not uniform and result in a variety of disaccharide units (4). There are 12 sulfation patterns of disaccharide units (Fig. 1), and these units have isoforms of HexA, *i.e.*, GlcA or IdoA. Combinations of these units enable the formation of disaccharide sequences specific for individual ligand proteins. Although the 3-*O*-sulfated units are usually less than 1% of total disaccharides (5), there are seven isoform genes of heparan sulfate 3-*O*-sulfotransferase (3OST) in our genome. This rare modification was first described as an essential component of the antithrombin (AT)-binding site (6). The 3OST-1, a specific enzyme for this modification, was purified and cloned as the first isoform of the gene family (7, 8). A number of reports related to the AT-binding 3-*O*-sulfate have been published to date because of its pharmacological importance. Unlike the 3OST-1, six other isoforms were searched out from the DNA database as a 3OST-1 homologue (9, 10, 11). It has been reported that the 3OST isoforms catalyze the formation of non-AT-binding 3-*O*-sulfates. Although accumulating evidence suggests the physiological importance of non-AT-binding 3-*O*-sulfates (12), the structure–function relationships of this modification remain obscure. The lack of a standard method to analyze this rare modification is hindering the research to clarify the biochemical functions of non-AT-binding 3-*O*-sulfates.

**Figure 1:**
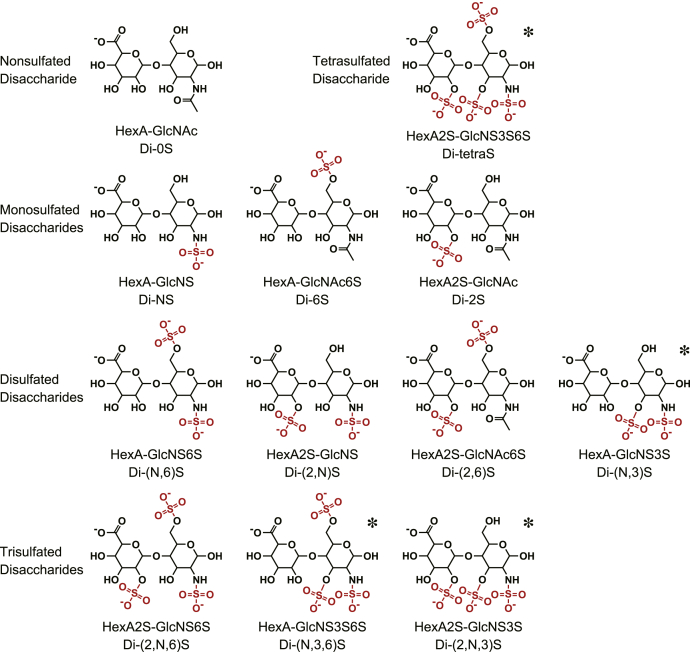
**Sulfation patterns of disaccharide units.** Twelve disaccharide units of heparan sulfate having differently sulfated motifs are illustrated, with the abbreviations used in this report. *Asterisks* indicate 3-*O*-sulfated units.

Previously, we analyzed a reaction product of 3OST isoforms and identified five 3-*O*-sulfated components, including three disaccharides and two tetrasaccharides, as a digestion product of heparin lyases (5, 13). The three disaccharides have been determined to be ΔHexA-GlcNS3S6S (Di-(N,3,6)S), ΔHexA2S-GlcNS3S (Di-(2,N,3)S), and ΔHexA2S-GlcNS3S6S (Di-tetraS) units. However, the two tetrasaccharides, named Tetra-1 and Tetra-2, have not yet been determined. Heparin lyases produced by *Flavobacterium heparinum* have been used for years to analyze heparan sulfate-related polysaccharides including heparin (14, 15). Using a mixture of three lyases (heparinase, heparitinase I, and heparitinase II), heparan sulfate is almost completely digested into unsaturated disaccharides with the exception of the AT-binding structure. The glucosaminidic linkage adjacent to GlcA-GlcNS3S ± 6S units, a critical component of the AT-binding site, is resistant to digestion and results in unsaturated tetrasaccharides (16). However, non-AT-binding 3-*O*-sulfated structures are susceptible to the lyases and digested into unsaturated disaccharides (5). Although there are a number of reports demonstrating the compositional analysis of heparan sulfate and heparin using heparin lyases, apart from our previous study, analysis of the 3-*O*-sulfated disaccharides has not been reported. Ours was the first to demonstrate the Di-tetraS unit as the most sulfated component of heparan sulfate. We also performed quantitative analysis of the Di-tetraS unit in heparan sulfate from various rat tissues and found rare but ubiquitous distribution of this unique structure (5, 13).

In this study, expanding our earlier work, we aimed to establish a method by which 3-*O*-sulfated components of heparan sulfate, especially non-AT-binding 3-*O*-sulfated units, can be quantified. Because the 3-*O*-sulfated tetrasaccharides have not been characterized, we first performed a structural analysis of the tetrasaccharides using a biochemical technique. We then prepared the five components as a standard saccharide for HPLC analysis. Together with non-3-*O*-sulfated disaccharides, the 13 units of heparan sulfate were analyzed by reverse-phase ion-pair HPLC using the postcolumn fluorescent labeling system. To test the method established here, we finally analyzed the compositional changes of 3-*O*-sulfated units in heparan sulfate from P19 cells before and after neuronal differentiation.

## Results

### Characterization of tetrasaccharides

**Figure 2:**
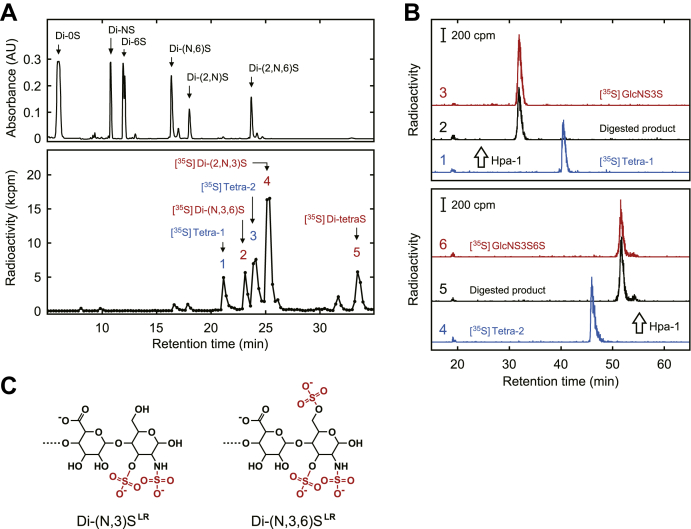
**Characterization of Tetra-1 and Tetra-2.***A*, preparation of 3-*O*-[^35^S]sulfated saccharides. Heparan sulfate (0.4 mg) was incubated with [^35^S]PAPS (1.1 × 10^7^ dpm) and 18 μg of recombinant human 3OST-5, at 30 °C for 6 h. The ^35^S-labeled heparan sulfate was digested with a mixture of heparin lyases and subjected to HPLC on a CarboPac PA1 column. The conditions for HPLC were as described in “Experimental Procedures.” The absorbance was monitored at 232 nm (*upper panel*). Six major components of heparan sulfate are indicated. Fractions of 0.2 ml were collected and aliquots used to measure the radioactivity (*lower panel*). Five radio-labeled products have been characterized in our previous study as indicated. The peak fractions were desalted and used for further analysis. *B*, HPLC analysis of ^35^S-labeled tetrasaccharides digested with Hpa-1. 3-*O*-[^35^S]Sulfated Tetra-1 and Tetra-2 (peaks *1* and *3* in *A*, respectively) were digested with Hpa-1 and analyzed by HPLC on a Carbo Pac PA1 column. The conditions for HPLC were as described in “Experimental Procedures.” *Upper panel* shows the analysis of Tetra-1. The radiochromatograms, *1* to *3*, show the untreated [^35^S]Tetra-1, the digested product, and [^35^S]GlcNS3S monosaccharide, respectively. *Lower panel* shows the analysis of Tetra-2. The radiochromatograms, *4* to *6*, show the untreated [^35^S]Tetra-2, the digested product, and [^35^S]GlcNS3S6S monosaccharide, respectively. [^35^S]GlcNS3S and [^35^S]GlcNS3S6S were prepared from [^35^S]Di-(2,N,3)S and [^35^S]Di-(N,3,6)S (peaks *4* and *2* in *A*, respectively) by treatment with mercuric acetate. *C*, reducing disaccharides of lyase-resistant tetrasaccharides. Tetra-1 and Tetra-2 were renamed as shown.

Since the Tetra-1 and Tetra-2 were expected to be derived from the AT-binding structure and have GlcA-GlcNS3S ± 6S units as a reducing disaccharide, we planned to characterize the tetrasaccharides by determining these units. We first prepared five 3-*O*-[^35^S]sulfated units in the same manner reported previously (13) and described in “Experimental Procedures.” ^35^S-labeled heparan sulfate prepared by incubating heparan sulfate with [^35^S]3'-phosphoadenosine 5'-phosphosulfate (PAPS) and recombinant human 3OST-5 was digested with a mixture of heparin lyases and separated by HPLC on a CarboPac PA1 column. When monitored at 232 nm, six major disaccharide units of heparan sulfate were identified (Fig. 2*A*, *upper panel*). The elution profile of radioactivity (*lower panel*) is consistent with our previous report, and five peaks have been characterized as follows. Peaks *2*, *4*, and *5* are Di-(N,3,6)S, Di-(2,N,3)S, and Di-tetraS, respectively. Peaks *1* and *3* are Tetra-1 and Tetra-2, respectively. The peak fractions were pooled and desalted for further analysis.

The ^35^S-labeled tetrasaccharides were digested with human heparanase-1 (Hpa-1) and analyzed by HPLC on a CarboPac PA1 column as described in “Experimental Procedures” (Fig. 2*B*). It has been reported that the Hpa-1 cleaves glucuronidic linkage of GlcA-GlcNS3S ± 6S units in the reducing end of heparan sulfate and releases GlcNS3S ± 6S monosaccharides (17). Nonsulfated GlcA is an essential residue of the cleavage reaction. The radio-chromatogram *1* shows the analysis of untreated [^35^S]Tetra-1. With the enzyme digestion, the retention time of ^35^S-labeled product was shifted to an earlier position (chromatogram *2*). Chromatogram *3* shows the analysis of [^35^S]GlcNS3S monosaccharide, prepared from [^35^S]Di-(2,N,3)S by treatment with mercuric acetate as described in “Experimental Procedures.” To confirm the molecular size, the digested product was also analyzed by HPLC on a gel filtration column. The ^35^S-labeled product was eluted at the position of [^35^S]GlcNS3S (data not shown). These data show that Tetra-1 has the GlcA-GlcNS3S unit as a reducing disaccharide. [^35^S]Tetra-2 was analyzed in the same way as above. The ^35^S-labeled product produced by the reaction of Hpa-1 was confirmed to be [^35^S]GlcNS3S6S monosaccharide by HPLC on a CarboPac PA1 column (Fig. 2*B*, *lower panel*) and a gel filtration column (data not shown). The results indicate that Tetra-2 has the GlcA-GlcNS3S6S unit as a reducing disaccharide. In consequence, the tetrasaccharides were renamed with the reducing disaccharides, *i.e.*, Di-(N,3)S^LR^ and Di-(N,3,6)S^LR^, where LR is lyase-resistant (Fig. 2*C*).

### Preparation of the 3-O-sulfated saccharide standards

**Figure 3:**
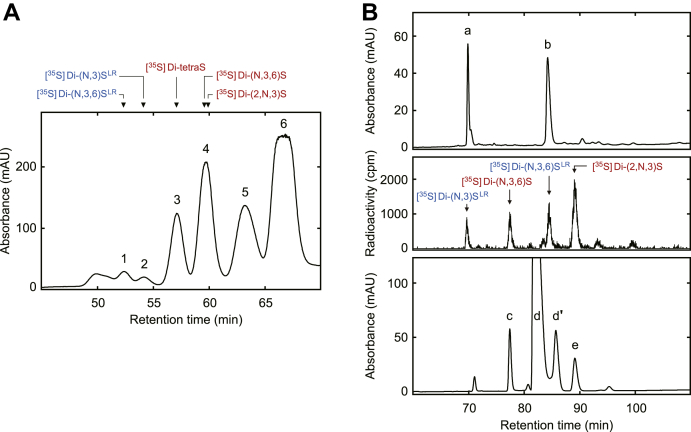
**Preparation of 3-*O*-sulfated saccharide standards.***A*, gel filtration chromatography of unsaturated saccharides derived from 3OST-5-modified heparan sulfate and heparin. Twelve milligrams of heparan sulfate and heparin were incubated with 80 μmol of PAPS and 1 mg of recombinant 3OST-5. The 3-*O*-sulfated products were then digested with a mixture of heparin lyases and subjected to HPLC on a Superdex Peptide 10/300 GL column. The conditions for HPLC were as described in “Experimental Procedures.” The absorbance was monitored at 232 nm, and peak fractions, corresponding to tetrasaccharides (peak *1* and *2*), tetrasulfated disaccharide (peak *3*), and trisulfated disaccharides (peak *4*), were pooled. *Arrow heads* indicate elution positions of ^35^S-labeled saccharides prepared in Figure 2*A*. *B*, separation of the 3-*O*-sulfated saccharides to a single component. Peak fractions from the gel filtration column were separated by HPLC on a CarboPac PA1 semipreparative column. The conditions for HPLC were as described in “Experimental Procedures.” *Upper* and *bottom panels* show the separation of tetrasaccharides and trisulfated disaccharides, respectively, monitored at 232 nm. The elution positions were confirmed by the ^35^S-labeled saccharides prepared in Figure 2*A* (*middle panel*). Peak fractions matched to each component were collected: Di-(N,3)S^LR^, peak *a*; Di-(N,3,6)S^LR^, peak *b*; Di-(N,3,6)S, peak *c*; Di-(2,N,3)S, peak *e*. Peaks *d* and *d'* are Di-(2,N,6)S, a major component of the heparin and its anomer.

3OST-5 produces the five 3-*O*-sulfated units from heparan sulfate and heparin in different proportions (5). To obtain sufficient amounts of all components, these substrates (12 mg of each) were sulfated with an excess amount of 3OST-5. The mixture of 3-*O*-sulfated products was then digested with a mixture of heparin lyases and separated by HPLC on a gel filtration column as described in “Experimental Procedures.” Figure 3*A* shows the elution profile monitored at 232 nm. The ^35^S-labeled saccharides prepared in “Characterization of Tetrasaccharides” were analyzed under the same conditions to confirm the elution positions of 3-*O*-sulfated saccharides, indicated by *arrow heads*. Because of insufficient separation, two tetrasaccharides (peaks *1* and *2*) were pooled as a single fraction in this step and separated by the CarboPac PA1 column as described below. Peak *3* is tetrasulfated disaccharide, *i.e.*, Di-tetraS. Peak *4* is trisulfated disaccharides containing Di-(N,3,6)S, Di-(2,N,3)S, and Di-(2,N,6)S. Peaks *5* and *6* are disulfated disaccharides and monosulfated disaccharides, respectively. The peak fractions containing 3-*O*-sulfated units were then separated into single components by HPLC on a CarboPac PA1 semipreparative column. *Upper* and *bottom panels* of Figure 3*B* show the separation of tetrasaccharides (the mixture of peaks *1* and *2* in *A*) and trisulfated disaccharides (peak *4* in *A*), respectively, monitored at 232 nm. To confirm the elution positions, the ^35^S-labeled saccharides were also analyzed under the same conditions (*middle panel*). Peaks *a* (Di-(N,3)S^LR^), *b* (Di-(N,3,6)S^LR^), *c* (Di-(N,3,6)S), and *e* (Di-(2,N,3)S) were pooled, desalted by a gel filtration column, and freeze-dried. Peaks *d* and *d'* are Di-(2,N,6)S and its anomer. The tetrasulfated disaccharide (peak *3* in *A*) was also applied to the CarboPac PA1 semipreparative column under the same conditions. A single peak fraction, eluted at the position of [^35^S]Di-tetraS, was pooled, desalted, and freeze-dried (data not shown). The freeze-dried saccharides were dissolved in 20 mM ammonium acetate buffer, pH 6.0. The standard saccharides were quantitated by measurement of the absorbance at 232 nm (18).

### Optimization of the reverse-phase ion-pair chromatography

**Figure 4:**
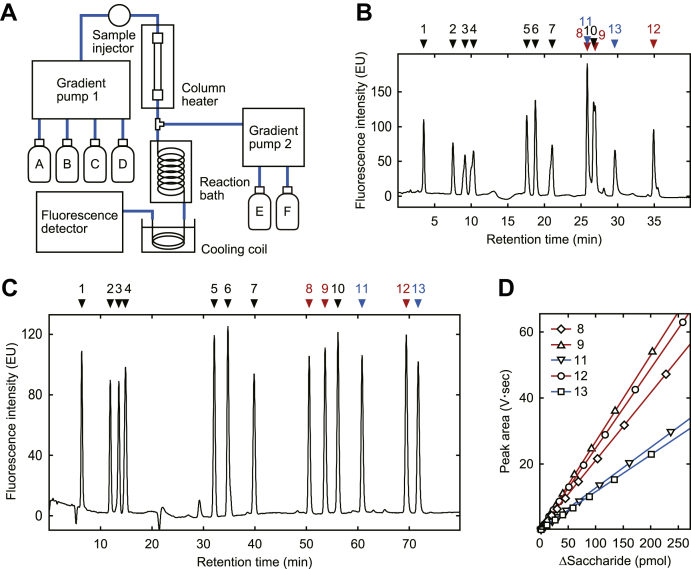
**Optimization of the reverse-phase ion-pair chromatography.***A*, reverse-phase ion-pair HPLC with the postcolumn fluorescent labeling system. The arrangement of the instruments and reagents is shown. *B*, chromatogram of the 13 standard units separated under previous conditions. The previous method was as follows. The eluants from A to D were distilled water, 0.4 M NaCl, 10 mM tetrabutylammonium bisulfate, and 50% acetonitrile, respectively. The column used was a DOCOSIL SP100 (4.6 × 150 mm; Senshu Scientific). A combination of linear gradients of eluant B was used, from 1 to 2% (0–10 min), 2 to 8% (10–11 min), 8 to 13% (11–20 min), 13 to 27% (20–22 min), 27% (22–29 min), 27 to 70% (29–33 min) followed by 70% (from 33 min). The proportions of eluants C and D remained constant at 12 and 17%, respectively. The flow rate was 1.1 ml/min, and the column temperature was 55 °C. Other reagents and labeling conditions were similar to those used for the optimized method. The peaks are Di-0S (*1*), Di-NS (*2*), Di-6S (*3*), Di-2S (*4*), Di-(N,6)S (*5*), Di-(2,N)S (*6*), Di-(2,6)S (*7*), Di-(N,3,6)S (*8*), Di-(2,N,3)S (*9*), Di-(2,N,6)S (*10*), Di-(N,3)S^LR^ (*11*), Di-tetraS (*12*), and Di-(N,3,6)S^LR^ (*13*). For abbreviations, see Figures 1 and 2*C*. *C*, chromatogram of the 13 standard units separated under optimized conditions. The optimized method is described in “Experimental Procedures.” *D*, standard curves of 3-*O*-sulfated saccharides. Linear correlations were observed as R^2^= 0.99992, 0.99995, 0.99989, 0.99981, and 0.99993 for *8*, *9*, *11*, *12*, and *13*, respectively.

Five 3-*O*-sulfated units, prepared in this study, and eight non-3-*O*-sulfated disaccharide units, obtained from Seikagaku Bio, were mixed as the standard mixture of 13 units. In the previous study, we employed reverse-phase ion-pair HPLC with a postcolumn fluorescent labeling system (Fig. 4*A*), described by Toyoda *et al.* (19, 20), in the analysis of the Di-tetraS unit. When the standard mixture was analyzed by the previous method, elution positions of 3-*O*-sulfated units overlapped with the Di-(2,N,6)S unit as shown in Figure 4*B*. Additionally, anomer separation of 3-*O*-sulfated units complicated the quantitative analysis. Thus we optimized the method to ensure accurate quantitative analysis of the 13 units. We tested gradient formations of eluant B, proportions of eluant C and D, water-soluble organic solvents for eluant D, pH adjustments of eluant C, and separation columns. As a result, we obtained a baseline separation of all components as shown in Figure 4*C*. The improved method is detailed in “Experimental Procedures.” The use of ethanol for eluant D and pH adjustment of eluant C to 8.0 are critical factors to reduce the anomer separation and result in a sharp symmetric peak. Under the optimized condition, the amounts of 3-*O*-sulfated units correlated with the integrated fluorescence intensity, as shown in Figure 4*D*. The standard curves of non-3-*O*-sulfated units were also obtained with linear correlations of R^2^ > 0.999 (data not shown).

### Analysis of heparan sulfate from P19 cells

**Figure 5:**
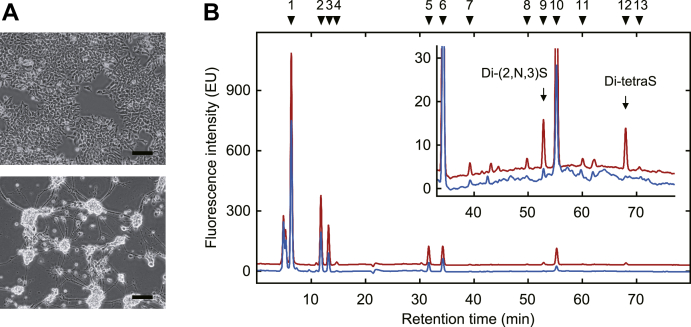
**Analysis of the heparan sulfate from P19 cells.***A*, typical photomicrographs of P19 cells and their differentiated neurons. P19 cells were cultured with α-MEM supplemented with 10% FBS (*Upper micrograph*). To induce neuronal differentiation, cells were seeded into a nontreated culture flask with α-MEM containing 2% FBS and 0.5 μM retinoic acid and cultured for 4 days to form the embryonic bodies. The aggregated cells were then dissociated into single cells and seeded into a poly-D-lysine coated flask with α-MEM containing 5% FBS and 30 μM cytarabine. The cells were cultured for the following 4 days. The differentiated neurons (*lower micrograph*) were harvested and homogenized with acetone. Scale bar; 100 μm. *B*, compositional analysis of the heparan sulfate derived from P19 cells and differentiated neurons. Heparan sulfate was prepared from 20 mg of dried acetone powders as described in “Experimental Procedures” and digested with a mixture of heparin lyases. The digested products were analyzed by HPLC with a postcolumn fluorescent labeling system. *Blue* and *red* chromatograms show the analysis of the digested products derived from undifferentiated and differentiated P19 cells, respectively. The *inset* represents an enlarged vertical axis with the same horizontal scale. The elution positions of standard saccharides are indicated by numbers; see legend to Figure 4.

Using the method established here, we examined the compositional changes of heparan sulfate from P19 cells before and after neuronal differentiation based on the evidence described in “Discussion.” P19 cells were differentiated into neurons by treatment with retinoic acid as described in “Experimental Procedures.” Heparan sulfates were prepared from P19 cells and differentiated neurons and digested with a mixture of heparin lyases. The digested products were analyzed by the HPLC with postcolumn fluorescent labeling method. Figure 5*A* shows the typical photomicrographs of P19 cells and differentiated neurons (*upper* and *lower panels*, respectively). Figure 5*B* shows representative chromatograms of the digested products derived from P19 cells (*blue line*) and differentiated neurons (*red line*). The *inset* represents an enlarged vertical axis with the same horizontal scale. Significant peaks of Di-(2,N,3)S and Di-tetraS were detected in the chromatogram derived from the neurons, as indicated by *arrows*, but not from the undifferentiated P19 cells. The molar percent of 13 components is shown in Table 1. The detection limit of the method was estimated to be 2 pmol of saccharides, ensured by the minimum points of standard curves in Figure 4*D*.

**Table 1:** Compositional analysis of heparan sulfate from P19 cells and their differentiated neurons (molar percent)

	Di-0S^a^	Di-NS	Di-6S	Di-2S	Di-(N,6)S	Di-(2,N)S	Di-(2,6)S	Di-(N,3,6)S	Di-(2,N,3)S	Di-(2,N,6)S	Di-(N,3)S^LR^	Di-tetraS	Di-(N,3,6)S^LR^
P19 cell	56 ± 0.7^b^	20 ± 0.5	6.0 ± 0.02	0.37 ± 0.13	4.2 ± 0.13	10 ± 0.2	0.10 ± 0.019	ND^c^	0.10 ± 0.008	3.4 ± 0.21	ND	ND	ND
Neuron	47 ± 1.9	21 ± 0.9	8.5 ± 0.17	0.99 ± 0.03	6.1 ± 0.18	8.4 ± 0.35	0.15 ± 0.009	0.07 ± 0.003	0.29 ± 0.007	7.0 ± 0.64	0.10 ± 0.002	0.29 ± 0.004	0.07 ± 0.002

a For abbreviations, see Figures 1 and 2C.

b Data represent means ± S.E. of three independent culture experiment.

c ND, not detected.

## Discussion

We established a method that quantifies 13 components of heparan sulfate, including three non-AT-binding and two AT-binding 3-*O*-sulfated units. The five 3-*O*-sulfated units have been identified as a reaction product of 3OST isoforms in our previous studies (5, 13). 3OST-1 produces Di-(N,3)S^LR^ and Di-(N,3,6)S^LR^ as a critical component of the AT-binding site. 3OST-2, 3OST-3, and 3OST-4 produce Di-(2,N,3)S and Di-tetraS as non-AT-binding structures. 3OST-5 has broad specificity and produces all five units, including Di-(N,3,6)S unit specific for this isoform. The lyase-susceptible Di-(N,3,6)S unit would be derived from IdoA-GlcNS3S6S and different from the reducing disaccharide of Di-(N,3,6)S^LR^. IdoA-aMan3S6S, where aMan represents 2,5-anhydromannose, and GlcA-aMan3S6S have been reported as the reaction products of 3OST-5 by nitrous acid degradation (21). It should be noted that 3OST-6, the youngest member of the 3OST gene family, has been cloned and characterized after our previous report. Xu *et al.* (11) reported that the 3OST-6 produces no AT-binding structures, and the reaction specificity of this isoform is similar to that of 3OST-3. Since our primary objective is non-AT-binding 3-*O*-sulfates, the preparation of tetrasaccharide standards was limited to two major components that account for 70% of the total reaction products of 3OST-1 (5), although other minor tetrasaccharides have been reported (16, 22). *N*-unsubstituted glucosamine residues have also been reported as a minor component of the heparan sulfate (23, 24). Improvement of the method to detect these minor components is the next step in achieving a comprehensive analysis of heparan sulfate.

We employed reverse-phase ion-pair HPLC as the separation method and aimed to determine the optimum conditions for efficient separation of the 13 units as described in “Results.” Consequently, a baseline resolution of all components was achieved. The detection limit of the postcolumn fluorescence labeling method is similar to that of mass spectrometry with a low pmol level (25). Although mass spectrometry is a powerful method for identifying the target molecule, application of the method is restricted to the HPLC system using volatile eluant. Therefore, the optimized HPLC conditions are unsuitable for mass spectrometry as is. A method by which all 13 units can be separated using a volatile buffer has yet to be established.

Huang *et al.* (26) reported the peeling reaction that specifically degrades heparan sulfate oligosaccharides having 3-*O*-sulfated glucosamine residue at the reducing end. This terminal residue is susceptible to degradation under mildly basic conditions (pH 8 and over), and temperature increases accelerate the reaction. We found that when the standard saccharides prepared in this study were dissolved in ammonium acetate buffer, pH 6.0, they could be stably stored at −80 °C for at least 1 year. In the compositional analysis, heparan sulfates were digested with the heparin lyases in a buffer solution of pH 7.0, at 30 °C for 4 h. To ensure stability of the digested products having 3-*O*-sulfated glucosamine residues, the standard mixture of 13 units was incubated under the same conditions. No quantitative changes and no degradation products were observed in the HPLC analysis (data not shown). Furthermore, we stop the lyase reaction by membrane filtration to remove the enzymes, rather than denaturation by heat.

We have previously analyzed the expression levels of 3OST isoforms in human tissue and found that the 3OST-1 and 3OST-3 are widely expressed in various tissues. In contrast, the 3OST-2 and 3OST-4 are specifically expressed in the brain. 3OST-5 is also a brain-specific isoform, although its expression level is relatively low. Moreover, it has been reported that expression of 3OST genes is spatiotemporally regulated in the developing brain of mouse and zebrafish (27, 28). Accordingly, we anticipated the 3-*O*-sulfated components specifically expressed in the neurons and selected P19 cells to test the method established here. To our knowledge, the compositional analysis of heparan sulfate, including the 3-*O*-sulfated units from P19 cells, has not yet been reported. In this study, we successfully detected Di-(2,N,3)S and Di-tetraS units in the heparan sulfate only from the differentiated neurons. More detailed studies on the temporal changes of 3-*O*-sulfated units related to the differentiation, the expression levels of 3OST genes in the differentiating cells, and the protein factors bound to the neuron specific 3-*O*-sulfated structures are the next step in the research to clarify the physiological roles and biochemical functions of 3-*O*-sulfated heparan sulfate in the neurons.

Although the structure–function relationships of non-AT-binding 3-*O*-sulfates are still obscure, accumulating evidence suggests the physiological importance of this modification. For example, the morphogenesis of mouse embryonic submandibular gland is regulated by the expression of 3OST-3 gene through FGF10/FGFR2b signaling (29). Borjigin *et al.* (30) demonstrated that the 3OST-2 is expressed in the rat pineal gland specifically during the daylight hours. Aberrant expressions of 3OST genes have been reported from various cancers, although the effects of 3OST genes are either antioncogenic or tumor-promoting (31). The importance of 3OST genes has also been reported using invertebrate model organisms. Hs3st-B is involved in the Notch signaling pathway in *Drosophila melanogaster* (32). In the nervous system of *Caenorhabditis elegans*, 3OST gene expressions are required for *kal-1*-dependent neurite branching (33). In most of these studies, the 3-*O*-sulfated components of heparan sulfate expressed in the target cells or tissues remain unknown. The lack of a regular method to analyze those rare components hinders the research. The method established here may provide a new approach to the physiological functions of non-AT-binding 3-*O*-sulfates. Twenty milligrams of acetone powder, obtained from two T75 flasks culturing semi-confluent P19 cells, was required for the compositional analysis of the 3-*O*-sulfated units. When we analyzed the heparan sulfate from rat tissues, the same amounts of acetone powders were sufficient to quantitate the 3-*O*-sulfated units (data not shown). Thus this method may be applied to most biological samples.

## Experimental procedures

### Materials

[^35^S]PAPS was purchased from PerkinElmer (Boston, MA). Heparin from porcine intestinal mucosa and all-trans retinoic acid were obtained from Sigma (St Louis, MO), PAPS from YAMASA (Tokyo, Japan), recombinant Hpa-1 from InSight (Rehovot, Israel), α-MEM and fetal bovine serum (FBS) from Gibco (Paisley, UK), cytarabine from Wako (Osaka, Japan), actinase E from KAKEN (Shizoka, Japan), and DNase I from Worthington (Lakewood, NJ). Heparan sulfate from bovine kidney, heparinase, heparitinase I, heparitinase II, and unsaturated disaccharide standards (Di-0S, Di-NS, Di-6S, Di-2S, Di-(N,6)S, Di-(2,N)S, Di-(2,6)S, and Di-(2,N,6)S) were obtained from Seikagaku Bio (Tokyo, Japan). Recombinant human 3OST-5 was expressed in insect cells and purified as reported previously (5).

### Preparation of 3-O-[^35^S]sulfated saccharides

The reaction mixture (0.2 ml) containing 50 mM Bis-Tris buffer, pH 6.0, 0.2 M NaCl, 2 mg/ml of heparan sulfate, 10 μM [^35^S]PAPS (1.1 × 10^7^ dpm), and 18 μg of recombinant human 3OST-5 was incubated at 30 °C for 6 h. Then ^35^S-labeled heparan sulfate was precipitated with two volumes of ethanol containing 1.3% (W/V) potassium acetate. After centrifugation, the precipitate was dissolved in 0.2 ml of water and reprecipitated to remove the remaining [^35^S]PAPS. The dried precipitate was dissolved in 65 μl of 40 mM sodium acetate buffer, pH 7.0, containing 4 mM CaCl_2_ and 0.4 mg/ml of bovine serum albumin (digestion buffer). The ^35^S-labeled heparan sulfate was digested with a mixture of heparinase (81 mU), heparitinase I (48 mU), and heparitinase II (32 mU) in 35 μl of digestion buffer at 30 °C for 5 h. After incubation, the reaction mixture was filtered with Nanosep 3K (PALL) to remove the enzymes and subjected to HPLC on a CarboPac PA1 column (4 × 250 mm; Thermo Scientific). A combination of linear LiCl gradients was used, from 0.03 to 0.12 M (0–5 min), 0.12 to 0.42 M (5–8 min), 0.42 to 0.81 M (8–15 min), 0.81 to 1.5 M (15–20 min), and 1.5 to 1.83 M (20–28 min) followed by 1.83 M (from 28 min). The flow rate was 0.8 ml/min, column temperature 40 °C. Absorbance was monitored at 232 nm. Fractions of 0.2 ml were collected, and aliquots were analyzed by liquid scintillation counting. Five radioactive peaks, corresponding to Tetra-1, Di-(N,3,6)S, Tetra-2, Di-(2,N,3)S, and Di-tetraS, were collected and applied to a Superdex Peptide 10/300 GL gel filtration column (1 × 30 cm, × 2 columns in series; GE Healthcare) to remove LiCl. The column was equilibrated and eluted with 0.1 M ammonium acetate buffer, pH 6.0, at a flow rate of 0.5 ml/min, at room temperature (26 °C). Fractions of 0.5 ml were collected, and aliquots were analyzed by liquid scintillation counting. The radioactive fractions were pooled and desalted by lyophilization. The dried [^35^S]saccharides were dissolved in 20 μl of water and used for further analysis.

### Preparation of 3-O-[^35^S]sulfated glucosamine monosaccharides

The 3-*O*-[^35^S]sulfated disaccharides (Di-(2,N,3)S and Di-(N,3,6)S) prepared in “Preparation of 3-*O*-[^35^S]sulfated saccharides” were treated with mercuric acetate to remove unsaturated hexuronic acid residues as described (34). The disaccharides were mixed with an equal volume of 70 mM mercuric acetate, pH 5.0, and incubated at room temperature for 10 min. The reaction products ([^35^S]GlcNS3S and [^35^S]GlcNS3S6S, respectively) were used as the monosaccharide standards.

### Structural analysis of the tetrasaccharides by digestion with Hpa-1

The 3-*O*-[^35^S]sulfated Tetra-1 and Tetra-2 prepared as described in “Preparation of 3-*O*-[^35^S]sulfated saccharides” were digested with 3.2 μg of Hpa-1 in 20 mM sodium acetate buffer, pH 5.0, containing 1 mM CaCl_2_. After the incubation at 30 °C for 24 h, the reaction mixture was filtered with Nanosep 3K and analyzed by HPLC on a CarboPac PA1 column (4 x 250 mm). A combination of linear LiCl gradients was used, from 0.03 to 0.12 M (0–10 min), 0.12 to 0.45 M (10–16 min), 0.45 to 0.9 M (16–30 min), 0.9 to 1.65 M (30–40 min), and 1.65 to 2.01 M (40–56 min) followed by 2.01 M (from 56 min). The flow rate was 0.4 ml/min, and the column temperature was 40 °C. The radioactivity was monitored with Beta-RAM flow scintillation detector (LabLogic). 3-*O*-[^35^S]sulfated glucosamine monosaccharide standards were also analyzed under the same conditions.

### Preparation of 3-O-sulfated standard saccharides

Twelve milligrams of heparan sulfate and heparin were incubated with 4 ml of reaction mixture containing 50 mM Bis-Tris buffer, pH 6.0, 0.4 M NaCl, 20 mM PAPS, and 1 mg of recombinant 3OST-5, at 30 °C for 96 h. The 3-*O*-sulfated saccharides were precipitated with two volumes of ethanol containing 1.3% (W/V) potassium acetate. The dried precipitates were dissolved in 1 ml of digestion buffer and digested with a mixture of heparinase (1.82 U), heparitinase I (1.09 U), and heparitinase II (0.73 U) at 30 °C for 48 h. In total, 0.1 volume of the digested products was separated by HPLC on a Superdex Peptide 10/300 GL column. The HPLC conditions were the same as described in “Preparation of 3-*O*-[^35^S]sulfated saccharides.” The eluate was monitored at 232 nm, and peak fractions, corresponding to tetrasaccharides, tetrasulfated disaccharide, and trisulfated disaccharides, were collected. The chromatography was repeated nine times. Fractions from each of nine columns were combined and freeze-dried. The dried samples were dissolved in 20 mM ammonium acetate buffer pH 6.0, then separated by HPLC on a CarboPac PA1 semipreparative column (9 × 250 mm). A combination of linear LiCl gradients was used, from 0 to 0.09 M (0–17 min), 0.09 to 0.33 M (17–27 min), 0.33 to 0.63 M (27–51 min), 0.63 to 1.14 M (51–68 min), 1.14 to 1.38 M (68–95 min), and 1.38 to 2.4 M (95–145 min). The flow rate was 1.2 ml/min, column temperature was 30 °C. Absorbance was monitored at 232 nm. Peak fractions having a retention time of 3-*O*-sulfated units were collected and desalted by a gel filtration column. The freeze-dried samples were dissolved in 20 mM ammonium acetate buffer, pH 6.0, and stored at −80 °C.

### Cell culture and differentiation

P19, mouse embryonic carcinoma cells, were purchased from the European Collection of Cell Culture. The cells were cultured and differentiated as previously described (35, 36). Briefly, the cells were grown and maintained in a T25 culture flask (CORNING) containing α-MEM supplemented with 10% FBS. To induce neuronal differentiation, cells were seeded into a nontreated T75 flask (eppendorf) at a density of 2.6 × l0^4^ cells per cm^2^ in α-MEM containing 2% FBS and 0.5 μM retinoic acid and cultured for 4 days to form the embryonic bodies, with medium replacement after 2 days. The aggregated cells were then dissociated into single cells by trypsinization and seeded into a poly-D-lysine coated T75 flask (CORNING) at 8.0 × l0^4^ cells per cm^2^ in α-MEM containing 5% FBS and 30 μM cytarabine. Cells were cultured for the following 4 days with medium replacement after 2 days. The differentiated neurons were used for the compositional analysis of heparan sulfate.

### Preparation of heparan sulfate from P19 cells

Cell layers in the T75 culture flask were rinsed three times with phosphate buffer saline and scraped into ice-cold acetone. The cell suspension was shaken vigorously and centrifuged. The cell pellet was resuspended with fresh acetone and homogenized using a POLYTRON homogenizer (KINEMATICA). The obtained powder was washed twice with acetone and dried under vacuum. Twenty milligrams of the dried powder was suspended in 0.6 ml of 0.1 M Tris buffer, pH 7.5, containing 4 mg of actinase E, and incubated at 50 °C for 20 h. After incubation, the reaction mixture was chilled and adjusted to pH 5.5 by the addition of 1.5 M sodium acetate buffer, pH 4.0. To the mixture, 1 mg of DNase I was added and incubated at room temperature for 3 h. After the incubation, the reaction mixture was precipitated with three volumes of ethanol containing 1.3% (W/V) potassium acetate. Centrifuged precipitate was dried under vacuum and dissolved in 240 μl of 0.4 M NaOH containing 0.3 M sodium borohydride. After incubation at room temperature for 20 h, pH of the solution was adjusted to 2.0 with 2 M HCl. Insoluble materials were removed by centrifugation, and the supernatant was neutralized with 1 M NaOH. Crude heparan sulfate was precipitated with three volumes of ethanol. Centrifuged precipitate was dissolved in 0.4 ml of water and dialyzed against water for 24 h. The dialyzed solution was freeze-dried and dissolved in 150 μl of 0.2 M ammonium acetate. To the solution, 0.1 ml of DEAE-sepharose (GE Healthcare) was added and agitated gently for 15 min. The sepharose was washed with 0.4 M ammonium acetate, followed by elution of heparan sulfate with 0.5 ml of 3 M ammonium acetate. The eluate was diluted with 5 ml of water and freeze-dried.

### Analysis of heparan sulfate by reverse-phase ion-pair HPLC with a postcolumn fluorescent labeling system

The freeze-dried heparan sulfates prepared from cultured cells were dissolved in 15 μl of 40 mM Bis-Tris buffer, pH 7.0, containing 4 mM CaCl_2_ and digested with a mixture of heparin lyases (6.0 mU of heparinase, 3.6 mU of heparitinase I, and 2.4 mU of heparitinase II) in 10 μl of the digestion buffer at 30 °C for 4 h. After incubation, the reaction mixture was filtered with Nanosep 3K. The digested products were separated by reverse-phase ion-pair HPLC on an L-column3 C18 (4.6 × 250 mm; CERI) and detected by a postcolumn fluorescent labeling method as described by Toyoda *et al.* (19, 20) with some modifications. The HPLC system was constructed with Alliance Separations Module (2695, Waters; including gradient pump 1 for the sample separation, sample injector and column heater), gradient pump 2 for fluorescent labeling reagents (600E, Waters), dry reaction bath (DB-5, Shimamura Instruments), and fluorescence detector (2475, Waters) as illustrated in Figure 4*A*. The eluants used were as follows: A, distilled water; B, 0.4 M NaCl; C, 10 mM tetrabutylammonium bisulfate; D, 50% ethanol. The eluant C was adjusted to pH 8.0 with 1 M NaOH. The proportion of eluant B was 0% (0–15 min) followed by linear gradient from 0 to 22% (15–80 min). The eluants C and D remained constant at 40 and 24%, respectively. The flow rate was 0.8 ml/min, and the column temperature was 45 °C. To the eluate, 59 mM 2-cyanoacetamide (reagent E) and 0.25 M NaOH (reagent F) were added at the same flow rate of 0.4 ml/min. The mixture was passed through a coiled peek tube (10 m × 0.5 mm I.D.) in a reaction bath at 125 °C and a following cooling coil (3 m × 0.25 mm I.D.) immersed in a water bath. The fluorescence was monitored with excitation at 346 nm and emission at 410 nm.

## Data availability

All data are contained within the article.

## Conflict of interest

The authors declare that they have no conflicts of interest with the contents of this article.
